# Comparative ICE Genomics: Insights into the Evolution of the SXT/R391 Family of ICEs

**DOI:** 10.1371/journal.pgen.1000786

**Published:** 2009-12-24

**Authors:** Rachel A. F. Wozniak, Derrick E. Fouts, Matteo Spagnoletti, Mauro M. Colombo, Daniela Ceccarelli, Geneviève Garriss, Christine Déry, Vincent Burrus, Matthew K. Waldor

**Affiliations:** 1 Channing Laboratory, Brigham and Women's Hospital, Harvard Medical School, Boston, Massachusetts, United States of America; 2 Department of Genetics, Tufts Medical School, Boston, Massachusetts, United States of America; 3 J. Craig Venter Institute, Rockville, Maryland, United States of America; 4 Dipartimento di Biologia Cellulare e dello Sviluppo, Universitá di Roma La Sapienza, Rome, Italy; 5 Centre d'Étude et de Valorisation de la Diversité Microbienne, Département de Biologie, Université de Sherbrooke, Sherbrooke, Québec, Canada; 6 Howard Hughes Medical Institute, Chevy Chase, Maryland, United States of America; Université Paris Descartes, INSERM U571, France

## Abstract

Integrating and conjugative elements (ICEs) are one of the three principal types of self-transmissible mobile genetic elements in bacteria. ICEs, like plasmids, transfer via conjugation; but unlike plasmids and similar to many phages, these elements integrate into and replicate along with the host chromosome. Members of the SXT/R391 family of ICEs have been isolated from several species of gram-negative bacteria, including *Vibrio cholerae*, the cause of cholera, where they have been important vectors for disseminating genes conferring resistance to antibiotics. Here we developed a plasmid-based system to capture and isolate SXT/R391 ICEs for sequencing. Comparative analyses of the genomes of 13 SXT/R391 ICEs derived from diverse hosts and locations revealed that they contain 52 perfectly syntenic and nearly identical core genes that serve as a scaffold capable of mobilizing an array of variable DNA. Furthermore, selection pressure to maintain ICE mobility appears to have restricted insertions of variable DNA into intergenic sites that do not interrupt core functions. The variable genes confer diverse element-specific phenotypes, such as resistance to antibiotics. Functional analysis of a set of deletion mutants revealed that less than half of the conserved core genes are required for ICE mobility; the functions of most of the dispensable core genes are unknown. Several lines of evidence suggest that there has been extensive recombination between SXT/R391 ICEs, resulting in re-assortment of their respective variable gene content. Furthermore, our analyses suggest that there may be a network of phylogenetic relationships among sequences found in all types of mobile genetic elements.

## Author Summary

Integrative and conjugative elements (ICEs) are a class of mobile genetic elements that are key mediators of horizontal gene flow in bacteria. These elements integrate into the host chromosome, yet are able to excise and transfer via conjugation. Our understanding of ICE evolution is rudimentary. Here, we developed a method to capture ICEs on plasmids, thus facilitating their sequencing. Comparative analyses of the DNA sequences of ICEs from the same family revealed that they have an identical genetic structure consisting of syntenous, highly conserved core genes that are interrupted by clusters of diverse variable genes. Unexpectedly, many genes in the core backbone proved non-essential for ICE transfer. Comparisons of the variable gene content in the ICEs analyzed revealed that these elements are mosaics whose genomes have been shaped by inter–ICE recombination. Finally, our work suggests that ICEs contribute to a larger gene pool that connects all types of mobile elements.

## Introduction

There are three types of self-transmissible mobile genetic elements: plasmids, bacteriophages and integrative conjugative elements (ICEs). All three classes of elements enable horizontal transmission of genetic information and all have had major impacts on bacterial evolution [1]–[4]. ICEs, (aka conjugation transposons), like plasmids, are transmitted via conjugation; however, unlike plasmids, ICEs integrate into and replicate along with the chromosome. Following integration, ICEs can excise from the chromosome and form circular molecules that are intermediates in ICE transfer. Plasmids and phages have been the subject of more extensive study than ICEs and while there is growing understanding of the molecular aspects of several ICEs [5]–[10], to date there have been few reports of comparative ICE genomics [11],[12] and consequently understanding of ICE evolution is only beginning to be unraveled.

Diverse ICEs have been identified in a variety of gram-positive and gram–negative organisms [13]. These elements utilize a variety of genes to mediate the core ICE functions of chromosome integration, excision and conjugation. In addition to a core gene set, ICEs routinely contain genes that confer specific phenotypes upon their hosts, such as resistance to antibiotics and heavy metals [14]–[18], aromatic compound degradation [19] or nitrogen fixation [20].

SXT is an ∼100 Kb ICE that was originally discovered in *Vibrio cholerae* O139 [16], the first non-O1 serogroup to cause epidemic cholera [21]. SXT encodes resistances to several antibiotics, including sulfamethoxazole and trimethoprim (which together are often abbreviated as SXT) that had previously been useful in the treatment of cholera. Since the emergence of *V. cholerae* O139 on the Indian subcontinent in 1992, SXT or a similar ICE has been found in most clinical isolates of *V. cholerae*, including *V. cholerae* serogroup O1, from both Asia and Africa. Other vibrio species besides *V. cholerae* have also been found to harbor SXT-related ICEs [22]. Furthermore, SXT-like ICEs are not restricted to vibrio species, as such ICEs have been detected in *Photobacterium damselae*, *Shewanella putrefaciens* and *Providencia alcalifaciens* [23]–[25]. Moreover, Hochhut et al [26] found that SXT is genetically and functionally related to the so-called ‘Inc J’ element R391, which was derived from a South African *Providencia rettgeri* strain isolated in 1967 [27]. It is now clear that Inc J elements are SXT-related ICEs that were originally misclassified as plasmids. In the laboratory, SXT has a fairly broad host range and can be transmitted between a variety of gram-negative organisms [16].

The SXT/R391 family of ICEs is now known to include more than 30 elements that have been detected in clinical and environmental isolates of several species of γ- proteobacteria from disparate locations around the globe [28]. SXT/R391 ICEs are grouped together as an ICE family because they all encode a nearly identical integrase, Int. Int, a tyrosine recombinase, is considered a defining feature of these elements because it enables their site-specific integration into the 5′ end of *prfC*, a conserved chromosomal gene that encodes peptide chain release factor 3 [29]. Int mediates recombination between nearly identical element and chromosome sequences, *attP* and *attB* respectively [29]. When an SXT/R391 ICE excises from the chromosome, Int, aided by Xis, a recombination directionality factor, mediates the reverse reaction - recombination between the extreme right and left ends (*attR* and *attL*) of the integrated element - thereby reconstituting *attP* and *attB* [6],[29]. The excised circular SXT form is thought to be the principal substrate for its conjugative transfer. The genes that encode activities required for SXT transfer (*tra* genes) were originally found to be distantly related to certain plasmid *tra* genes [30]–[32]. The *tra* genes encode proteins important for processing DNA for transfer, mating pair formation and generating the conjugation machinery. Regulation of SXT excision and transfer is at least in part governed by a pathway that resembles the pathway governing the lytic development of the phage lambda. Agents that damage DNA and induce the bacterial SOS response are thought to stimulate the cleavage and inactivation of SetR, an SXT encoded λ cI-related repressor, which represses expression of *setD* and *setC*, transcription activators that promote expression of *int* and *tra* genes [5].

The complete nucleotide sequences of SXT (99.5kb) and R391 (89kb) were the first SXT/R391 ICE family genomes to be reported [14],[32]. Comparative [33] and functional genomic analyses [5],[32] revealed that these 2 ICEs share a set of conserved core genes that mediate their integration/excision (*int* and *xis*), conjugative transfer (various *tra* genes), and regulation (*setR*, *setCD*). In addition to the conserved genes, these 2 ICEs contain element specific genes that confer element specific properties such as resistance to antibiotics or heavy metals. Interestingly, many of these genes were found in identical locations in SXT and R391, leading Beaber et al [33] to propose that there are ‘hotspots’ where SXT/R391 ICEs can acquire new DNA. The genomes of two additional SXT/R391 ICEs, ICE*Pda*Spa1, isolated from *Photobacterium damselae* [23], and ICE*Spu*PO1, derived from an environmental isolate of *Shewanella putrefaciens* [24] are now also known. These two genomes also share most of the conserved set of core genes present in SXT and R391 and contain element specific DNA.

Determination of the sequences of SXT/R391 family ICE genomes was a fairly arduous task due to their size and predominantly chromosomal localization. Here, we developed a method to capture and then sequence complete SXT/R391 ICE genomes. In addition, we identified 3 as yet unannotated SXT/R391 ICE genomes in the database of completed bacterial genomes. Comparative analyses of the 13 SXT/R391 genomes now available allowed us to greatly refine our understanding of the organization and conservation of the core genes that are present in all members of this ICE family. Comparative and functional analyses also facilitated our proposal of the minimal functional SXT/R391 ICE genome. Furthermore, this work provides new knowledge of the considerable diversity of genes and potential accessory functions encoded by the variable DNA found in these mobile elements. Finally, this comparative genomics approach has allowed us to garner clues regarding the evolution of this class of mobile elements.

## Results/Discussion

### An ICE capture system

**Figure 1:**
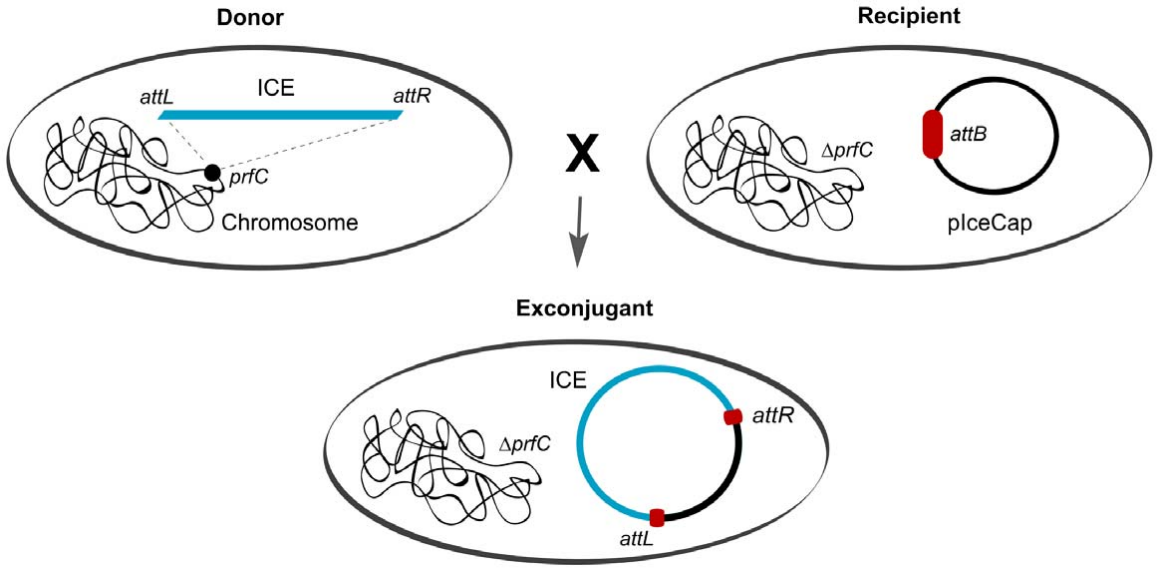
Schematic of the ICE capture system. Conjugation between a donor strain bearing a chromosomal ICE and a ΔprfC recipient strain harboring pIceCap, which contains *attB*, yields exconjugants that contain the transferred ICE integrated into pIceCap. Exconjugants were selected for using a marker on pIceCap and on the ICE. *attR* and *attL* represent the right and left ICE-chromosome junctions.

To date, ICE sequencing has been cumbersome because it has typically required construction of chromosome-derived cosmid libraries and screening for sequences that hybridize to ICE probes [23],[32]. We constructed a vector (pIceCap) that enables capture of complete SXT/R391 ICE genomes on a low-copy plasmid to simplify the protocol for ICE sequencing. This plasmid is a derivative of the single-copy modified F plasmid pXX704 [34],[35], which contains a minimal set of genes for F replication and segregation but lacks genes enabling conjugation. We modified pXX704 to include an ∼400bp fragment that encompasses the SXT/R391 attachment site (*attB*) and thereby enabled Int-catalyzed site-specific recombination between *attB* on pIceCap and *attP* on an excised and transferred ICE to drive ICE capture (Figure 1). Conjugations between an SXT/R391 ICE-bearing donor strain and an *E. coli* recipient deleted for *prfC* (and thus chromosomal *attB*) and harboring pIceCap yielded exconjugants containing the transferred ICE integrated into pIceCap (Figure 1). We used the Δ*prfC* recipient to bias integration of the transferred ICE into pIceCap rather than the chromosome. In these experiments, we selected for exconjugants containing the transferred ICE integrated into pIceCap, using an antibiotic marker present on the ICE as well as a marker present in pIceCap. The low copy IceCap::ICE plasmid was then isolated and used as a substrate for shotgun sequencing. We also found that the IceCap::ICE plasmids were transmissible. Thus, in principle this technique should facilitate capture of ICEs that do not harbor genes conferring resistance to antibiotics, by mating out the IceCap::ICE plasmid into a new recipient and selecting for the marker on pIceCap.

### SXT/R391 ICEs included in this analysis

A list of the 13 SXT/R391 ICEs whose genomes were analyzed and compared in this study is shown in Table 1. All of the ICEs included in our analyses contain an *int* gene that was amplifiable using PCR primers for *int_sxt_* [29]. They were isolated on 4 continents and from the Pacific Ocean during a span of more than 4 decades. They are derived from 7 different genera of γ-proteobacteria and the ICEs derived from *V. cholerae* strains are from both clinical and environmental isolates of 3 different *V. cholerae* serogroups.

**Table 1:** SXT/R391 ICE family members analyzed in this study.

ICE	Host strain	Site and year of isolation	Size (bp)	% Identity to Int*_SXT_*	Resistance profile	Notable Variable Genes	Genbank Accession Number	Strain or ICE References
ICE*Vch*Mex1	*Vibrio cholerae* non O1-O139	San Luis Potosi, Mexico 2001	82839	99% (410/413)	*-*	Fic family protein, diguanylate cyclase, restriction modification system	*GQ463143*	[66]
ICE*Vch*Ind4	*Vibrio cholerae* O139	Kolkata, India 1997	95491	100% (413/413)	*floR, strBA, sul2*	Toxin-antitoxin system	*GQ463141*	[54]
ICE*Vch*Ind5	*Vibrio cholerae* O1	Sevagram, India 1994	97847	99% (409/413)	*floR, strBA, sul2, dfrA1*	AraC family transcription regulator, glyoxoylase abx resistance	*GQ463142*	This study
ICE*Vch*Ban5	*Vibrio cholerae* O1	Bangladesh, 1998	102131	99% (409/413)	*floR, strBA, sul2, dfrA1*	AraC family transcription regulator, glyoxoylase abx resistance	*GQ463140*	[29]
ICE*Pal*Ban1	*Providencia alcalifaciens*	Bangladesh, 1999	96586	99% (409/413)	*floR, strBA, sul2, dfrA1*	Toxin-antitoxin system, phenazine biosynthesis protein, lysine exporter	*GQ463139*	[25]
ICE*Vfl*Ind1	*Vibrio fluvialis*	Kolkata, India 2002	91369(a)	99% (409/413)	*dfr18, floR, strBA, sul2*	Toxin-antitoxin system	*GQ463144*	[22]
ICE*Vch*Moz10	*Vibrio cholerae* O1	Beira, Mozambique 2004	104495	99% (409/413)	*floR, strBA, sul2, tetA'*	AraC family transcription regulator, glyoxoylase abx resistance, ATP-dependent Lon protease	*ACHZ00000000*	[67]
ICE*Pmi*Usa1	*Proteus mirabilis*	Maryland, United States 1986	79733	99% (409/413)	*-*	ATP-dependent helicase	*AM942759*	[68]
ICE*Vch*Ban9	*Vibrio cholerae* O1	Matlab, Bangladesh 1994	106124	99% (409/413)	*floR, strBA, sul2, dfrA1, tetA'*	AraC family transcription regulator, glyoxoylase abx resistance, ATP-dependent Lon protease	*CP001485*	[69]
ICE*Vch*Ban8	*Vibrio cholerae* non O1-O139	Bangladesh, 2001	105790(a)	25% (76/301)	*-*	Toxin-antitoxin system	*NZ_AAUU00000000*	This study
SXT^MO10^	*Vibrio cholerae* O139	Chennai, India 2002	99452	100%	*dfr18, floR, strBA, sul2*	Toxin-antitoxin system	*AY055428*	[16]
R391	*Providencia rettgeri*	Pretoria, South Africa 1967	88532	99% (410/413)	*kanR, merRTPCA*	Sulfate transporter, universal stress protein	*AY090559*	[27]
ICE*Pda*Spa1	*Photobacterium damselae*	Galicia, Spain 2003	102985	99% (412/413)	*tetAR*	ATP-dependent Lon protease, heat-shock protein	*AJ870986*	[23]
ICE*Spu*PO1	*Shewanella putrefaciens*	630m, Pacific Ocean 2000	108623	99% (409/413)	-	Zn/Co/Cd efflux system, restriction modification system	*CP000503*	[24]

(a) The sequence is not complete and therefore the true size is not known.

Five of these ICE genome sequences were determined at the J. Craig Venter Institute (JCVI) using the ICE capture system described above (Table 1, rows 1–5). In addition, we sequenced ICE*Vfl*Ind1, also at the JCVI, by isolating cosmids that encompassed this *V. fluvialis* derived ICE prior to developing the ICE capture technique (Table 1, row 6). Table 1 (rows 7–10) also includes 4 previously unannotated ICE genomes that we found in BLAST searches of the NCBI database of completed but as yet unannotated genomes; 3 of these ICEs are clearly members of SXT/R391 ICE family since they are integrated into their respective host's *prfC* locus and contain *int* genes that are predicted to encode Int proteins that are 99% identical to Int*_sxt_*. The fourth element, ICE*Vch*Ban8 does not encode an Int*_sxt_* orthologue; however, this element contains nearly identical homologues of most of the known conserved core SXT/R391 ICE family genes. ICE*Vch*Ban8 will be discussed in more detail below but since it does not contain an Int*_sxt_* orthologue it is not considered a member of the SXT/R391 family of ICEs and thus not included in our comparative study. Finally, Table 1 also includes the 4 SXT/R391 ICEs that were previously sequenced (Table 1, rows 11–14).

Despite the diversity of our sources for SXT/R391 ICEs, the genomes of two pairs of ICEs that we analyzed proved to be very similar. SXT^MO10^ and ICE*Vch*Ind4 only differed by 13 SNPs in 7 genes and by the absence from ICE*Vch*Ind4 of *dfr18*, a gene conferring trimethoprim resistance. These ICEs were derived from *V. cholerae* O139 strains isolated in India from different cities at different times: SXT^MO10^ from Chennai in 1992 and ICE*Vch*Ind4 from Kolkata in 1997. The high degree of similarity of these two ICE genomes suggests that ICEs can be fairly stable over time. ICE*Vch*Ban9 and ICE*Vch*Moz10 were also extremely similar although ICE*Vch*Moz10 lacks *dfrA1*, another allele for trimethoprim resistance. These two ICEs were derived from *V. cholerae* O1 strains from Bangladesh (1994) and Mozambique (2004) respectively. The great similarity of these ICEs suggests that there has been spread of SXT-related ICEs between Asia and Africa in recent times. Studies of CTX prophage genomes have also suggested the spread of *V. cholerae* strains between these continents [36].

### General structure and sizes of SXT/R391 genomes

**Figure 2:**
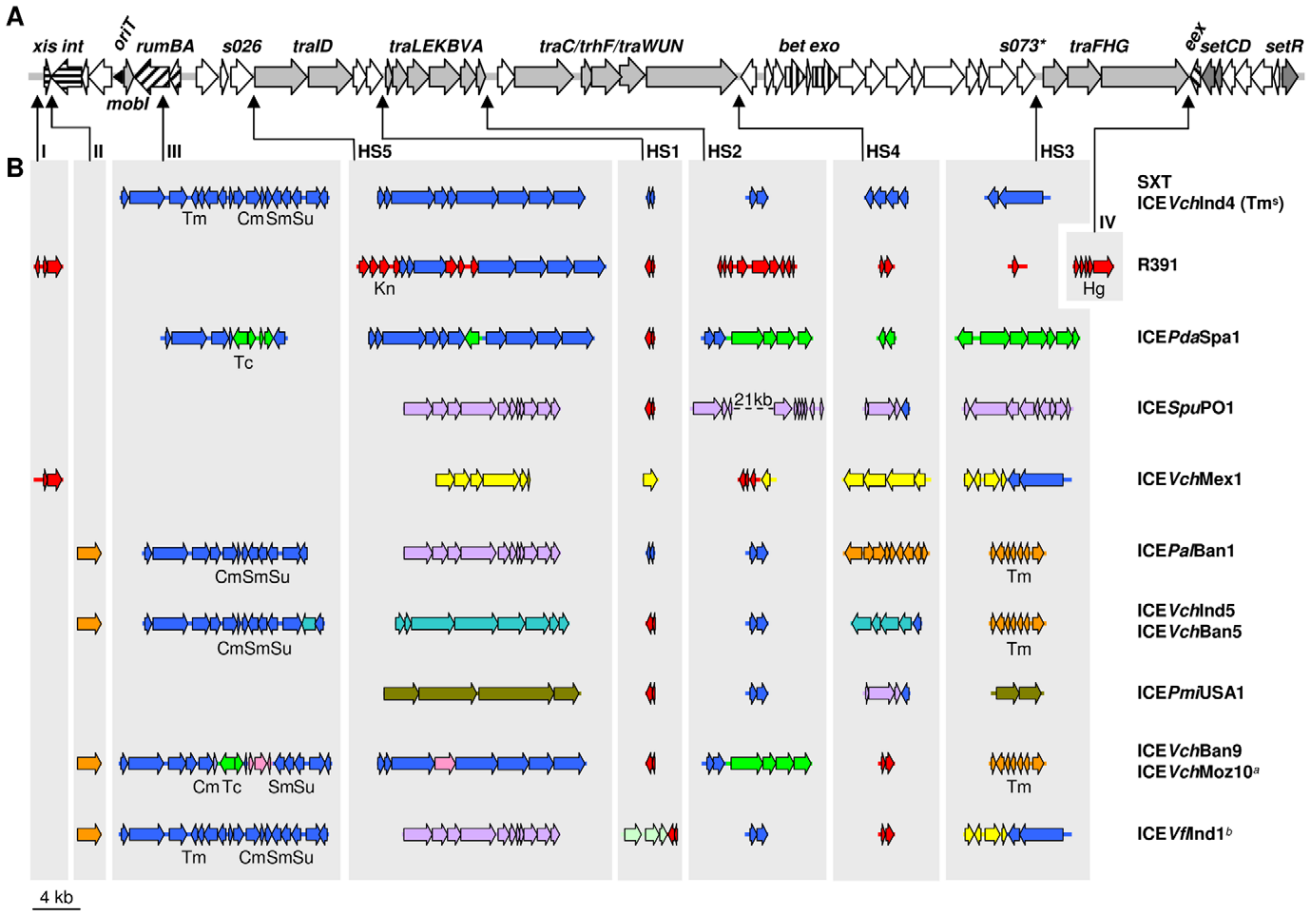
Structure of the genomes of 13 SXT/R391 ICEs. (A) The upper line represents the set of core genes (thick arrows) and sequences common to all 13 SXT/R391 genomes analyzed. Hatched ORFs indicate genes involved in site-specific excision and integration (*xis* and *int*), error-prone DNA repair (*rumAB*), DNA recombination (*bet* and *exo*) or entry exclusion (*eex*). Dark gray ORFs correspond to genes involved in regulation (*setCDR*). Light gray ORFs represent genes encoding the conjugative transfer machinery, and white ORFs represent genes of unknown function. (B) Variable ICE regions are shown with colors according to the elements in which they were originally described SXT (blue), R391 (red), ICE*Pda*Spa1 (green), ICE*Spu*PO1 (purple), ICE*Vch*Mex1 (yellow), ICE*Pal*Ban1 (orange), ICE*Vch*Ind5 (turquoise), ICE*Pmi*USA1 (olive), ICE*Vch*Ban9 (pink), ICE*Vfl*Ind1 (light green). Thin arrows indicate the sites of insertion for each variable region and HS1–HS5 represent hotspots 1–5. Roman numerals indicate variable regions not considered true hotspots. Cm, chloramphenicol; Hg, mercury; Kn, kanamycin; Sm, streptomycin; Su, sulfamethoxazole; Tc, tetracycline; Tm, trimethoprim. * indicates that *s073* is absent from ICE*Pda*Spa1. *^a^* ICE*Vch*Moz10, which lacks *dfrA1* in the integron structure, does not confer resistance to Tm. *^b^* The purple gene content of ICE*Vfl*Ind1 was deduced from partial sequencing, PCR analysis and comparison with ICE*Spu*PO1.

The ICEs listed in Table 1 were initially compared using MAUVE [37] and LAGAN [38], programs that enable visualization of conserved and variable regions on a global scale. All of the SXT/R391 ICEs we analyzed share a common structure and have sizes ranging from 79,733 bp to 108,623 bp (Table 1 and Figure 2). They contain syntenous sets of 52 conserved core genes (Figure 2A) that total approximately 47kb and encode proteins with an average of 97% identity to those encoded by SXT. All of the individual ICEs also contain DNA that is relatively specific for individual elements (Figure 2B); the differences in the sizes of the variable regions accounts for the range in ICE sizes.

Five sites within the conserved SXT/R391 ICE structure have variable DNA present in all of the ICEs in Figure 2. Four of these sites were previously termed ‘hotspots’ for ICE acquisition of new DNA [33]. Due to similarities between SXT and R391, the fifth hotspot only became apparent through our comparison of the 13 ICEs examined here. Each of these hotspots (HS1 to HS5 in Figure 2B) is found in an intergenic region (see below), suggesting that the acquisition of these variable DNA regions has not interrupted core ICE gene functions. In addition, some of the ICEs have variable DNA inserted in additional intergenic locations or in *rumB* (labeled I–IV in Figure 2B). Previous analyses [32] indicated that the insertion in *rumB*, did not impair SXT transmissibility. Overall, comparison of these 13 SXT/R391 ICE genomes suggests that: 1) these elements consist of the same perfectly syntenous and nearly identical 52 core genes that serve as a scaffold (see below) capable of mobilizing a large range of variable DNA; and 2) selection pressure to maintain ICE mobility has restricted insertions of variable DNA into sites that do not interrupt core functions.

### The SXT/R391 ICE core genes

The 52 core genes present in all the SXT/R391 ICEs analyzed include sets of genes that are known to be required for the key ICE functions of integration/excision, conjugative transfer and regulation [32] as well as many genes of unknown function. Most genes of known or putative (based on homology) function (coded by gray shading or hatch marks in Figure 2A) are clustered with genes that have related functions. For example, *int* and *xis*, genes required for integration and excision, are adjacent and *setR*, and *setC/D*, the key SXT regulators are near each other at the extreme 3′ end of the elements, although separated by 4 conserved genes of unknown function. Each ICE also has four gene clusters implicated in conjugative DNA processing and transfer (shown in light gray in Figure 2A). Finally, each of the ICEs has a nearly identical origin of transfer (*oriT*), a cis-acting DNA site that is thought to be nicked to initiate DNA processing events during conjugative transfer [39], in the same relative location.

The conserved core genes include approximately as many genes of unknown function as genes of known function. Some of the genes of unknown function are found either interspersed amongst gene clusters that likely comprise functional modules (e.g *s091* between *traD* and *s043*) while others are grouped together (e.g. most genes between *traN* and *traF*). In several cases, the interspersed genes appear to be part of operons with genes of known function (e.g. *s086-s082* maybe in an operon with *setDC*).

### Variable ICE DNA

In addition to sharing 52 core genes, all of the ICE genomes analyzed contain variable DNA regions, ranging in size from 676 to 29,210 bp. Most of the variable DNA sequences are found in 5 intergenic hotspots (Figure 2B). However, some ICEs contain additional variable DNA inserts outside the 5 hotspots. For example, SXT and five other ICEs in Figure 2 have variable DNA segments, corresponding to related IS*CR2* elements, disrupting *rumB* (Figure 2B, site III). IS*CR2* elements are IS*91*-like transposable elements that tend to accumulate antibiotic resistance genes [40]. Interestingly, it is unusual for the contents of the hotspots and other variable regions to be found in only one ICE. Instead, the variable gene content of most of the ICEs shown in Figure 2B is found in more than one ICE. For example, ICE*Spu*PO1, ICE*Pal*Ban1, and ICE*Vfl*Ind1, all have identical contents in hotspot 5 (lavender genes in hotspot 5 in Figure 2B); however, the contents of the other hotspots in these 3 elements are almost entirely different. Thus, the variable gene content of the SXT/R391 ICEs reveals that these elements are mosaics. The overlapping distribution of variable DNA segments seen in the ICEs in Figure 2B suggests that recombination among this family of mobile elements may be extensive. In addition, in some instances, the variable regions appear subject to additional genetic modifications. For example, ICE*Pda*Spa1 and ICE*Vch*Ban9 contain ICE-specific DNA nested within the shared sequences inserted at hotspot 5 DNA (the green and pink genes in hotspot 5 in these elements, Figure 2B).

The variable genes encode a large array of functions and only a few will be discussed here. A complete list of the diverse genes found in the hotspots is found in Table S1. Although we cannot predict functions for many genes found in the hotspots, since they lack homology to genes of known function, at least a subset of the known genes seem likely to confer an adaptive advantage upon their hosts. Most of the ICE antibiotic resistance genes are found within transposon-like structures (e.g., the ISCR2 elements noted above) but four ICEs contain a *dfrA1* cassette, which confers resistance to trimethoprim [25], in a class IV integron located in hotspot 3. A disproportionate number of variable genes are likely involved in DNA modification, recombination or repair, as they are predicted to encode diverse putative restriction-modification systems, helicases and endonucleases. Such genes may provide the host with barriers to invasion by foreign DNA including phage infection and/or promote the integrity of the ICE genome during its transfer between hosts. Three ICEs contain genes that encode diguanylate cyclases [41] in hotspot 3. These enzymes catalyze the formation of cyclic-diguanosine monophosphate (c-di-GMP), a second messenger molecule that regulates biofilm formation, motility and virulence in several organisms including *V. cholerae* [42],[43]. Most SXT/R391 ICEs contain *mosA* and *mosT* in hotspot 2. These two genes encode a novel toxin-antitoxin pair that promotes SXT maintenance by killing or severely inhibiting the growth of cells that have lost this element [44]. Not all ICEs in the SXT/R391 family contain *mosAT*; however, those lacking these genes may encode similar systems to prevent ICE loss. For instance, R391 and ICE*Vch*Mex1 contain two genes (*orf2* and *orf3*) encoding a predicted HipA-like toxin and a predicted transcriptional repressor distantly related to the antitoxin HipB.

### Locations of the ICE variable genes

**Figure 3:**
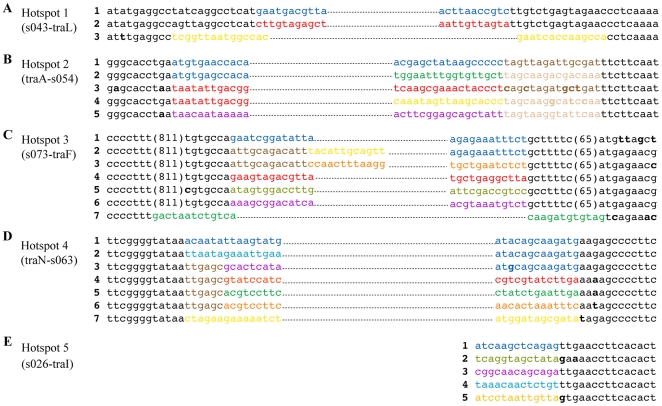
The boundaries of the 5 hotspots. The boundaries between conserved and hotspot variable regions are shown. Black typeface indicates conserved sequence, while color indicates variable sequence. Numbers in parentheses indicate the number of intervening nucleotides. The thin dotted lines indicate continuations of variable DNA. Bold letters indicate a non-conserved base. (A) Hotspot 1, which is present between *traJ* and *traL*. Line 1: SXT, ICE*Vch*Ind4, ICE*Pal*Ban1; Line 2: R391, ICE*Pda*Spa1, ICE*Vch*Ban5, ICE*Vch*Ind5, ICE*Pmi*USA, ICE*Spu*PO1, ICE*Vfl*Ind, ICE*Vch*Moz10, ICE*Vch*Ban9; Line 3: ICE*Vch*Mex1. (B) Hotspot 2, which is present between *traA* and *s054*. Line 1: SXT, ICE*Vch*Ind4, ICE*Pmi*USA, ICE*Vfl*Ind, ICE*Vch*Ind5, ICE*Pal*Ban1, ICE*Vch*Ban5; Line 2: ICE*Pda*Spa1, ICE*Vch*Moz10, ICE*Vch*Ban9; Line 3: R391; Line 4: ICE*Vch*Mex1; Line 5: ICE*Spu*PO1. (C) Hotspot 3, which is present between *s073* and *traF*. Line 1: SXT, ICE*Vch*Ind4; Line 2: ICE*Vch*Mex1, ICE*Vfl*Ind; Line 3: ICE*Vch*Moz10, ICE*Vch*Ban9, ICE*Vch*Ind5, ICE*Pal*Ban1, ICE*Vch*Ban5. Line 4: R391; Line 5: ICE*Pmi*USA; Line 6: ICE*Spu*PO1; Line 7: ICE*Pda*Spa1. (D) Hotspot 4, which is present between *traN* and *s063*. Line 1: SXT, ICE*Vch*Ind4. Line 2: ICE*Vch*Ind5, ICE*Vch*Ban5; Line 3: ICE*Spu*PO1, ICE*Pmi*USA; Line 4: R391, ICE*Vch*Moz10, ICE*Vch*Ban9, ICE*Vfl*Ind. Line 5: ICE*Pda*Spa1; Line 6: ICE*Pal*Ban1; Line 7: ICE*Vch*Mex1. (E) Hotspot 5, which is present between *s026* and *traI*. Line 1: SXT, ICE*Vch*Ind4, ICE*Pda*Spa1, R391, ICE*Vch*Moz10, ICE*Vch*Ban9; Line 2: ICE*Pmi*USA; Line 3: ICE*Spu*PO1, ICE*Pal*Ban1, ICE*Vfl*Ind1; Line 4: ICE*Vch*Ind5, ICE*Vch*Ban5; Line 5: ICE*Vch*Mex1.

The variable regions found in the 5 hotspots are found exclusively in intergenic regions, punctuating the conserved ICE backbone (Figure 2). The boundaries between the conserved and variable sequences were mapped on the nucleotide level and compared (Figure 3A–3E). Each hotspot had a distinct boundary. Remarkably, even though the contents of the variable regions markedly differ, with few exceptions the left and right boundaries between conserved and variable DNA for each hotspot was identical among all the ICEs (Figure 3). For example, the left junctions of the inserts in hotspot 2 immediately follow the stop codon of *traA* and the right junctions are exactly 79 bp upstream of the start of *s054* (Figure 3B), despite the fact that the DNA contents within these borders greatly differ. In hotspot 2, the right junction appears to begin with a 15 bp sequence that has two variants (Figure 3B, brown & light brown sequence). These sequences may reflect the presence of earlier insertions that have since been partially replaced. A similar pattern was found adjacent to the left boundary of hotspot 4 in several ICEs (Figure 3D, lines 3–6). Once an insertion is acquired, the number of permissive sites for the addition of new variable DNA likely increases.

There are two exceptions to the precise boundaries between variable and conserved DNA. Hotspot 1 and hotspot 3 in ICE*Vch*Mex1 and ICE*Pda*Spa1, respectively, contain variable DNA that extends beyond the boundary exhibited by all the other ICEs in these locations (Figure 3A, line 3, and Figure 3C, line 7). The only boundary that could not be identified was the left border of hotspot 5, the region containing genes between *s026* and *traI*. As discussed below, *s026* is the least conserved core gene and its variability obscured any consensus sequence abutting the variable DNA. Perhaps this border has eroded because *s026* is not required for ICE mobility [32].

The relative precision of most boundaries between conserved and variable DNA sequences in all the ICEs analyzed suggests that a particular recombination mechanism, such as *bet*/*exo*-mediated recombination, may explain the acquisition of the variable regions. However, at this point, we cannot exclude the possibility that the precise locations for variable DNA insertions simply reflects selection for optimal ICE fitness; i.e., ICEs can optimally accommodate variable DNA in these locations while preserving their essential functions.

### Similarity of SXT/R391 ICE and IncA/C plasmid core genes

**Figure 4:**
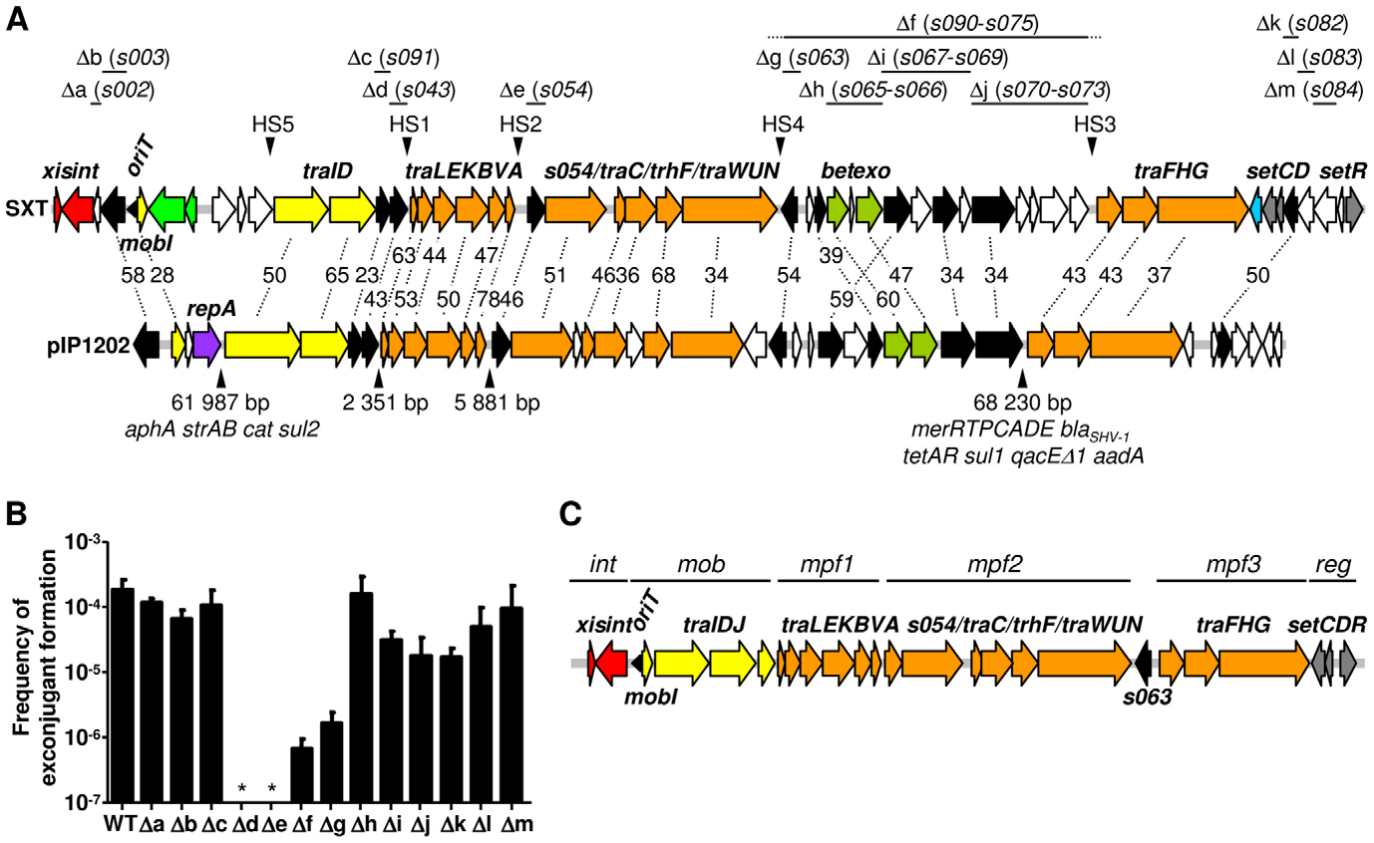
Comparison of the SXT/R391 core genome with the genome of pIP1202 and defining the minimal functional SXT/R391 gene set. (A) Alignment of the conserved core genes of SXT/R391 ICEs with the genome of the IncA/C conjugative plasmid pIP1202 from *Yersinia pestis*. The top line shows the same core ICE genes shown in Figure 2A. ORFs are color coded as follows: DNA processing, yellow; mating pair formation, orange; DNA recombination and repair, green; integration/excision, red; replication, purple; regulation, gray; entry exclusion, blue; homologous genes of unknown function, black; genes without corresponding counterparts in ICEs and pIP1202, white. Numbers shown in the middle represent % identity between the orthologous proteins encoded by SXT and pIP1202 [GenBank:NC_009141]. The positions of the hotspots in SXT/R391 ICEs are marked by downward pointing arrowheads. For pIP1202, the size of the sequences (which include IncA/C backbone DNA as well as variable DNA) found at these locations as well as resistance markers are indicated by upward pointing arrowheads. *aphA*, *aadA* and *strAB* confer resistance to aminoglycosides. *sul1* and *sul2* confer resistance to sulfonamides. *cat*, *bla_SHV-1_*, *tetAR*, *qacED1* and *merRTPCADE* confer resistance to chloramphenicol, β-lactams, tetracyclines, quaternary ammonium compounds and mercury ions, respectively. Detailed descriptions of the conserved backbone of the IncA/C conjugative plasmids have been published elsewhere [48],[50]. Regions that were deleted from SXT to investigate the function of genes of unknown function (see panel B) are indicated with straight lines. Dotted lines indicate that the deletion included DNA in the adjacent hotspots. (B) Influence of deletion of genes of unknown function on the frequency of SXT transfer. The mean values and standard deviations from three independent experiments are shown. * indicates that the frequency of transfer was below the detection level (<10^−8^). Deletion mutants SXTΔa, SXTΔk and SXTΔl, transferred at frequencies that were not significantly different from that of wild-type SXT (data not shown). (C) Proposed minimal set of genes necessary for a functional SXT/R391 ICE. *int*, integration/excision module; *mob*, DNA processing module; *mpf*, mating pair formation modules; *reg*, regulation module.

Unexpectedly, BLAST analyses revealed that most of the conserved core SXT/R391 genes are also present in IncA/C conjugative plasmids. These multidrug resistance plasmids are widely distributed among *Salmonella* and other enterobacterial isolates from agricultural sources [45],[46]. Recently, members of this family of plasmids have also been identified in *Yersinia pestis*, including from a patient with bubonic plague [47], and in aquatic γ-proteobacteria [48], including *Vibrio cholerae* [49],[50]. To date, the closest known relatives of the SXT/R391 transfer proteins are found in the IncA/C plasmids. Every predicted SXT transfer protein is encoded by the IncA/C plasmid pIP1202 isolated from *Y. pestis* [50] and the identities of these predicted protein sequences vary from 34 to 78% (Figure 4A). Furthermore, there is perfect synteny between the four gene clusters encoding the respective conjugative machineries of these two mobile elements (yellow and orange genes in Figure 4A). Despite the extensive similarity of the SXT and IncA/C conjugative transfer systems, these plasmids lack homologues of *setR* and *setD/C* as well as *int/xis*, suggesting that regulation of conjugative transfer differs between these elements.

The similarity of IncA/C plasmids and SXT/R391 ICEs is not limited to genes important for conjugal DNA transfer. Ten genes of unknown function (shown in black in Figure 4A), some of which are interspersed within likely *tra* gene operons and some of which are clustered together between *traN* and *traF*, are similar in the two elements. Furthermore, most of these ten genes are in identical locations in the two elements. Both elements also contain homologs of *bet* and *exo* (shown in green in Figure 4A); these are the only known homologs of the λ Red recombination genes found outside of bacteriophages. Together, the similarity of DNA sequences and organization of SXT/R391 ICEs and IncA/C plasmids suggests that these elements have a common ancestor. The fact that the contents of the hotspots in the two classes of elements are entirely distinct suggests that their evolutionary paths diverged prior to acquisition of these variable DNA segments.

### The minimal functional SXT/R391 ICE gene set

The conservation of the 52 core genes in all 13 SXT/R391 ICEs analyzed suggested that many or even all of these genes would be required for key ICE functions of excision/integration, conjugative transfer and regulation. The presence of ten ICE core genes of unknown function in IncA/C plasmids (black genes in Figure 4A) is also consistent with the hypothesis that these genes might be required for ICE transfer. However, our previous work demonstrated that not all genes recognized here as part of the conserved core gene set are required for SXT transfer. Beaber et al showed that deletion of *rumB – s026* (which includes 5 cores genes) from SXT had no detectable influence on SXT excision or transfer [32]. Therefore, we systematically deleted all of the core ICE genes whose contributions had not previously been assessed, in order to explore the hypothesis that these genes (especially those also present in IncA/C plasmids) would be essential for ICE transfer and to define the minimum functional SXT/R391 gene set.

Surprisingly, deletion of most of the ICE core genes of unknown function, including genes with homologues in IncA/C plasmids, did not alter SXT transfer efficiency. Deletion of *s002* or *s003*, which are located downstream of *int* in all SXT/R391 ICEs, did not alter the frequency of SXT transfer; similarly, deletion of *s082*, *s083*, and *s084*, core genes of unknown function that are found near the opposite end of SXT/R391 ICEs but not in IncA/C plasmids, also did not influence SXT transfer frequency (Figure 4B). Furthermore, deletion of *s091*, which is found between *traD* and *s043* in ICEs and IncA/C plasmids, did not reduce SXT transfer (Figure 4B). In contrast, deletion of *s043*, which has weak homology to *traJ* in the F plasmid (a gene important in DNA processing) and is located in a transfer cluster containing *traI* and *traD*, abolished transfer (Figure 4B, Δd), suggesting that *s043*, here re-named *traJ* is required for SXT transfer. It is unlikely that the transfer defect of SXTΔ*traJ* can be explained by polar effects of the deletion on downstream genes, since *traJ* appears to be the last gene of an operon found immediately upstream of hotspot 1. Similarly, deletion of *s054*, which is found immediately 5′ of *traC* and is homologous to a disulfide-bond isomerase *dsbC*, also abolished transfer (Figure 4B, Δe). Interestingly, disulfide bond-isomerases are present in several other conjugative systems [51]. However, it is not clear at this point if the deletion of *s054* from SXT accounts for the transfer defect of SXTΔ*s054*, since we could not restore transfer by complementation.

Additionally, Beaber et al found that deletion of *s060* through *s073* in SXT, which includes 7 genes that are also found in IncA/C plasmids reduced SXT transfer more than 100-fold [32]. We constructed several smaller deletions in this region and found that deletion of *s063*, which is also found in pIP1202, reduced the transfer frequency of SXT by ∼100-fold, nearly the same amount as deleting the entire region (Figure 4B). Complementation analyses revealed that the absence of *s063* accounted for the transfer defect of SXTΔ*s063* (data not shown). Even though SXTΔ*s063* was still capable of transfer, in our view, the drastic reduction in the transfer frequency of this mutant warrants inclusion of *s063* into the minimum functional SXT ICE genome (shown in Figure 4C). Other deletions in this region, including deletions of *bet*, *exo*, *s067*, *s068* and *s070*, which have orthologues in IncA/C plasmids, resulted in ≤10-fold reductions in transfer frequency. We therefore did not include these genes in the minimal functional core SXT/R391 genome (Figure 4C).

The findings from our experiments testing the transfer frequencies of SXT derivatives harboring core gene deletions (shown in Figure 4B), coupled with our previous work demonstrating the requirements for the predicted SXT *tra* genes in the element's transfer [32], suggest a minimal functional SXT/R391 ICE structure as shown in Figure 4C. This minimum element is ∼29.7 kb and consists of 25 genes. Genes with related functions, which in some cases encode proteins that likely form large functional complexes (such as the conjugation apparatus), are grouped together in the minimal genome. At the left end of the minimum ICE genomes are *xis* and *int*, the integration/excision module of SXT/R391 ICEs. In the minimal ICE genome, the ICE *oriT* and *mobI*, which encodes a protein required for SXT transfer [39], are no longer separated from the other genes (*traIDJ*) that are also thought to play roles in the DNA processing events required for conjugative DNA transfer. The genes required for formation of the conjugation machinery, including the pilus, and mating pair formation and stabilization [32],[39] are divided between three clusters (denoted mpf1-3 in Figure 4C). Finally, at the right end of the minimal functional genome are the genes that regulate ICE transfer (*setC/D* and *setR*). Thus, the minimal functional SXT/R391 ICE is relatively small and organized into 3 discrete functional modules that mediate excision/integration, conjugation, and regulation.

Even though deletion of 27 out of 52 SXT/R391 ICE core genes proved to have little or no effect on SXT transfer frequency, and hence these genes were not included in Figure 4C, it is reasonable to presume that these genes encode functions that enhance ICE fitness given their conservation. For example, the presence of highly conserved *bet* and *exo* genes in all SXT/R391 ICEs suggests that there has been selection pressure to maintain this ICE-encoded recombination system that promotes ICE diversity by facilitating inter ICE recombination (G Garriss, MK Waldor, V Burrus, in press). A key challenge for future studies will be to determine how core genes of unknown function promote ICE fitness.

### Variations in the similarity of core genes

**Figure 5:**
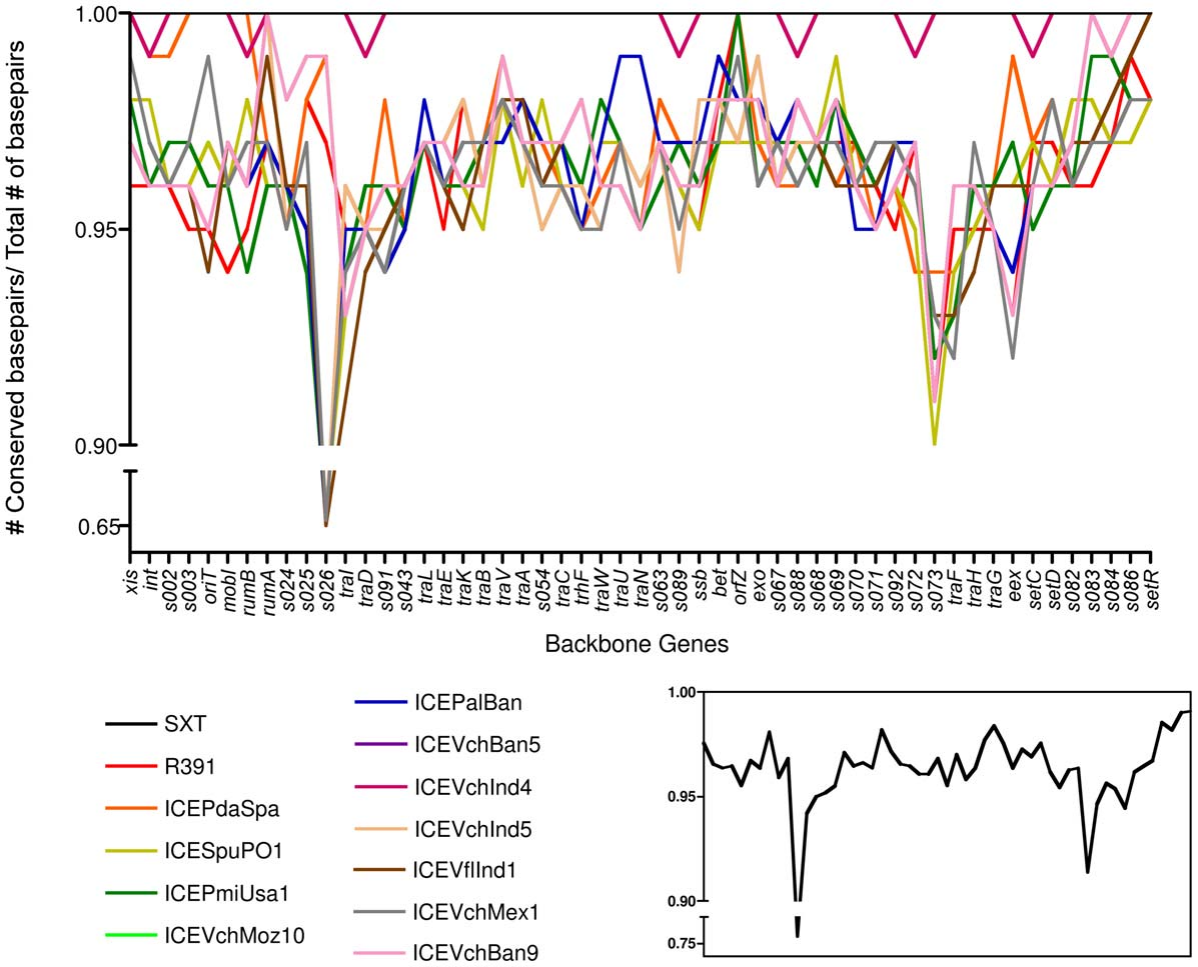
Variations in the nucleotide conservation of core ICE genes. The nucleotide sequence of each core gene from each ICE was compared to the corresponding sequence in SXT using pairwise BLASTn analyses to determine the percent identities. The average values for all of the ICEs, excluding SXT and ICE*Vch*Ind4, are shown in the inset.

To identify genes in the SXT/R391 core genome that may be subject to different selection pressures, we compared the percent identity of each ICE's core genes to the corresponding SXT gene (Figure 5). Most of the ICEs' core genes exhibited 94% to 98% identity on the nucleotide level to SXT's core genes. There was no discernable difference in the degree of conservation of most core genes that were or were not part of the minimal ICE, suggesting that there are equal selective pressures on essential and non-essential genes. However, we identified 8 genes (*s026*, *traI*, *orfZ*, *s073*, *traF*, *eex*, *s086*, and *setR*) that exhibit significantly different degrees of conservation (Figure 5 and Figure S1). Three of these showed unusually high conservation, while the other 5 had below average conservation. Two of the highly conserved genes, *setR* and *s086*, are found at the extreme 3′ end of the elements. The conservation of *setR* may reflect the key role of this gene in controlling SXT gene expression. S086 may also play a role in regulating SXT transfer [52]. The other highly conserved gene, *orfZ*, is found between *bet* and *exo* and has no known function.

*s026* and *s073* are the most divergent of all the genes in the backbone. *s026* encodes a hypothetical protein with homologues in many gram negative organisms. Although S026 is predicted to contain a conserved domain, COG2378, which has a putative role in transcription regulation, this protein is not required for SXT transfer [32]. The significant divergence of *s026* along with its lack of essentiality suggests that this gene could become a pseudogene. A similar argument could be made for *s073*, which encodes a hypothetical protein that is also not required for ICE transfer. However, this argument does not hold for *traI* or *traF*, two genes which are essential for ICE transfer. Although the reasons which account for the different degrees of conservation of these 8 core genes are hard to ascertain at this point, the data in Figure 5 suggests that individual core genes are subject to different evolutionary pressures.

### Comparisons of core gene phylogenies

**Figure 6:**
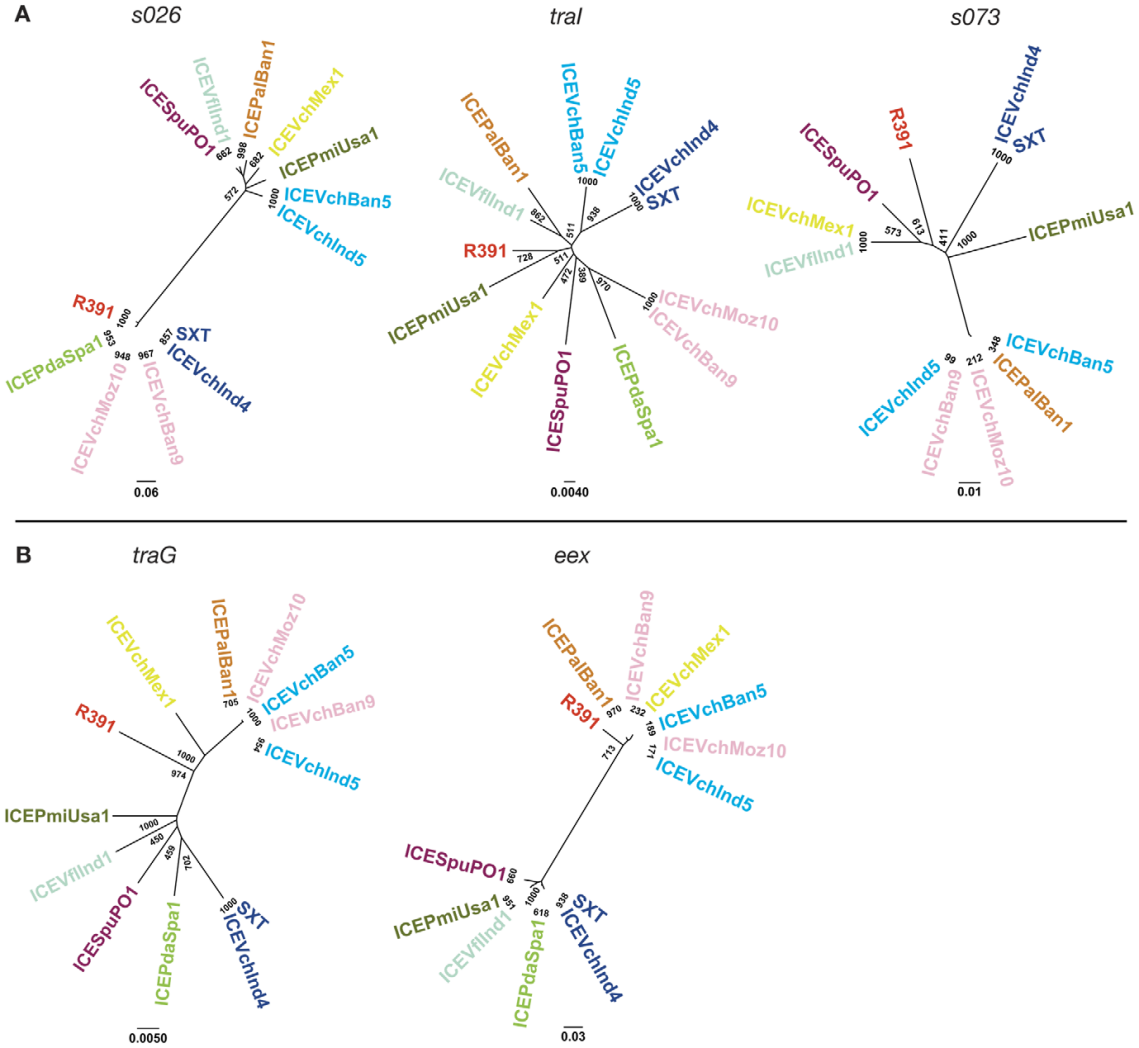
Phylogenetic analysis of several core ICE genes. Nucleotide sequences of the indicated core genes were used to generate the phylogenetic trees shown. Bootstrap values are indicated at branch points. The individual scale bars represent genetic distances and reflect the number of substitutions per residue.

We created phylogenetic trees for each core gene based on their respective nucleotide sequences to further explore the evolution of the conserved backbone of SXT/R391 ICEs. Since we found such a high degree of conservation for most of the core genes, the bootstrap values for most of these trees were relatively low. Thus, we concentrated on the most polymorphic genes found in Figure 5, *s026*, *s073*, *traI*, and *eex*, for phylogenetic analyses. As shown in Figure 6A, the trees for *s026*, *traI* and *s073* exhibit 3 distinct branching patterns. The lack of similarity in these phylogenetic trees suggests that either individual core genes have evolved independently or that high degrees of recombination mask their common evolutionary history. The latter hypothesis seems more likely since experimental findings have revealed that SXT/R391 ICEs can co-exist in a host chromosome in tandem [26] and recombination between tandem elements can yield novel hybrid ICEs with considerable frequency [53] (G Garriss, MK Waldor, V Burrus, in press). Also, as noted above, the distributions of variable genes among the ICEs shown in Figure 2 also supports the idea that inter-ICE recombination is commonplace.

Unlike most core genes, the trees for *traG* and *eex* were similar. In these two trees, the ICEs segregate into two evolutionarily distinct groups (Figure 6B), confirming and extending previous observations that revealed that there are two groups of *eex* and *traG* sequences in SXT/R391 ICEs [54]. These two groups correspond to the two functional SXT/R391 ICE exclusion groups. Interactions between *traG* and *eex* of the same group mediate ICE exclusion [55]. Thus, the identical 2 clusters of *traG* and *eex* sequences observed in their respective trees reveals the co-evolution of the *traG*/*eex* functional unit. The two groups of *eex* sequences can also be observed in Figure 5 where the bifurcating pattern reveals the 2 exclusion groups. This pattern is difficult to discern for *traG*, perhaps because of the large size of this multi-functional gene.

### ICE*Vch*Ban8, an SXT-like ICE that lacks Int*_sxt_*

The sequence of ICE*Vch*Ban8, which was derived from a non-O1, non-O139 *V. cholerae* strain, is incomplete but it appears to contain 49 out of 52 SXT/R391 core genes. However, since this strain lacks Int*_sxt_* it was not included in our comparative analyses above. It is not known if ICE*Vch*Ban8 is capable of excision or transmission; however, it contains a P4-like integrase and a putative *xis*. It is tempting to speculate that the genome of ICE*Vch*Ban8 provides an illustration of how acquisition (presumably via recombination) of a new integration/excision module may generate a novel ICE family.

### Perspectives

Comparative analysis of the genomes of the 13 SXT/R391 ICEs studied here has greatly refined our understanding of this group of mobile genetic elements. These elements, which have been isolated from 4 continents and the depths of the Pacific Ocean, all have an identical genetic structure, consisting of the same syntenous set of 52 conserved core genes that are interrupted by clusters of diverse variable genes. All the elements have insertions of variable DNA segments in the same five intergenic hotspots that interrupt the conserved backbone. Furthermore, some of the elements have additional insertions outside the hotspots; however, in all cases the acquisition of variable DNA has not compromised the integrity of the core genes required for ICE mobility. Functional analyses revealed that less than half of the conserved genes are necessary for ICE transmissibility and the contributions of the 27 core genes of unknown function to ICE fitness remains an open question. Finally, several observations presented here suggest that recombination between SXT/R391 ICEs has been a major force in shaping the genomes of this widespread family of mobile elements.

Although comparisons of the 13 ICE genomes analyzed here strongly suggest that these mobile elements have undergone extensive recombination during their evolutionary histories, there is a remarkable degree of similarity among the SXT/R391 ICEs. All of these ICEs consist of the same syntenous and nearly identical 52 genes. In contrast, other families of closely related mobile elements, such as lambdoid or T4-like phages for example, exhibit greater diversity [56],[57]. Since the elements that we sequenced were isolated from several different host species and from diverse locations, the great degree of similarity of the SXT/R391 ICE family does not likely reflect bias in the elements that we sequenced. It is possible that this family of mobile elements is a relatively recent creation of evolution and has yet to undergo significant diversification.

To date, relatively few formal comparative genomic analyses of other ICE families have been reported. Mohd-Zain et al [11] identified several diverse ICEs and genomic islands that shared a largely syntenous set of core genes with ICE*Hin*1056, an ICE originally identified in *Haemophilus influenzae*. However, even though these elements share a similar genomic organization, they exhibit far greater variability in the sites of insertion of variable DNA and in the degree of conservation in their core genes compared to SXT/R391 ICEs. Thus, although this group of elements appears to share a common ancestor, they seem to have diverged earlier in evolutionary history than the SXT/R391 ICEs. However, when comparative genomic analyses were restricted to ICE*Hin*1056-related ICEs found in only two *Haemophilus sp.*, Juhas et al found that, like the SXT/R391 family of ICEs, these 7 ICE*Hin*1056-related ICEs share greater than 90% similarity at the DNA level in their nearly syntenous set of core genes [12]. It will be interesting to learn the extent of conservation of genetic structure and DNA sequence in additional ICE families to obtain a wider perspective on ICE evolution.

Comparative genomic studies of bacteriophages have led to the idea that the full range of phage sequences are part of common but extremely diverse gene pool [58],[59]. The SXT/R391 ICE genomes suggest that there may be an even larger network of phylogenetic relationships linking sequences found in all types of mobile genetic elements including phages, plasmids, ICEs and transposons. The genomes of SXT/R391 ICEs appear to be amalgams of genes commonly associated with other types of mobile elements. Many of the ICE core genes are usually associated with phages, such as *int*, *bet*, *exo* and *setR*, or with plasmids, such as the *tra* genes. Additionally, the SXT/R391 ICEs and IncA/C plasmids clearly have a common ancestor, as we found that the entire set of SXT/R391 *tra* genes are also present in IncA/C plasmids. Thus, the genes present in all types of mobile genetic elements appear to contribute to a common gene pool from which novel variants of particular elements (such as ICE*Vch*Ban8) or perhaps even novel types of mobile genetic elements can arise.

## Materials and Methods

### ICE Sequencing

ICE*Pal*Ban1, ICE*Vch*Mex1, ICE*Vch*Ind4, ICEVchInd5 and ICE*Vch*Ban5 were isolated using the plasmid capture system described in Figure 1. The SXT chromosomal attachment sequence, *attB*, was introduced into the modified F plasmid pXX704 [34] to create pIceCap. This plasmid was then introduced into a Δ*prfC* derivative of the Tc^R^ *E. coli* strain CAG18439. Exconjugants derived from matings between this strain and those harboring the 5 ICEs listed above resulted in strains carrying a pIceCap::ICE plasmid. Once captured, the plasmids were isolated using the Qiagen plasmid midi kit for low-copy plasmids (Qiagen). Isolated pIceCap::ICE plasmids were then sequenced.

ICE*Vfl*Ind genome was determined by sequencing several overlapping cosmids that encompassed this ICE's genome. Briefly, genomic DNA from a *Vibrio fluvialis* strain carrying ICE*Vfl*Ind was prepared using the GNome DNA kit (QBIOgene). Sau3A1 restricted genomic DNA was used to create a SuperCos1 (Stratagene)-based cosmid library according the manufacture's instructions. The library was subsequently screened for cosmids containing ICE-specific sequences using PCR with primers to conserved core ICE sequences. Four cosmids containing overlapping ICE*Vfl*Ind sequences were identified and sequenced.

The genomes of 6 ICEs were sequenced by the Sanger random shotgun method [60]. Briefly, small insert plasmid libraries (2–3 kb) were constructed by random nebulization and cloning of pIceCap::ICE DNA or of cosmid DNA for ICE*Vfl*Ind. In the initial random sequencing phase, 8–12 fold sequence coverage was achieved. The sequences of either pIceCap or pSuperCos were subtracted and the remaining sequences were assembled using the Celera Assembler [61]. An initial set of open reading frames (ORFs) that likely encode proteins was identified using GLIMMER [62], and those shorter than 90 base pairs (bp) as well as some of those with overlaps eliminated.

### Bioinformatics

Nucleotide and amino acid conservation were assessed with the appropriate BLAST algorithms. ICEs were aligned using clustalW with default settings [63]. MAUVE [37] and LAGAN [38] were used to identify core genes in Figure 2. To map the boundaries of the hotspots, sequence comparisons were made using MAUVE and then manually compared to find boundaries between conserved and variable DNA as shown in Figure 3.

Phylogenetic trees were generated from alignments of nucleotide sequences using the neighbor-joining method as implemented by ClustalX software, version 2.011 [64]. The reliability of each tree was subjected to a bootstrap test with 1000 replications. Trees were edited using FigTree 1.22 (http://tree.bio.ed.ac.uk/software/figtree/).

### Generation and testing of SXT deletion mutants

CAG81439 harboring SXT was used as the host strain to create the SXT deletion mutants shown in Figure 3; the deletions were constructed using one-step gene inactivation as previously described [44],[65]. The primers used to create the deletion mutants are available upon request. Matings were conducted as previously described [16],[44] using deletion mutants and a Kn^R^ *E. coli* recipient, CAG18420. Exconjugants were selected on LB agar plates containing chloramphenicol, 20µg/ml (for SXT selection) and kanamycin, 50 µg/ml. The frequency of exconjugant formation was calculated by dividing the number of exconjugants by the number of donors.

## Supporting Information

Figure S1 Variations in the conservation of individual core ICE genes. The percent identity of the nucleotide sequence of each core gene and *oriT* versus the corresponding sequence in SXT was calculated for all ICEs studied. The average values for each gene (as shown in the inset of Figure 5) were then used in one-way ANOVA comparisons to determine genes that exhibit significantly more or less conservation compared to other core genes. p-values of one-way ANOVA comparisons of each core ICE gene are shown. The grid represents all pair-wise comparisons, and the color indicates the level of significance as follows: red: p<.001, orange: p<.01, and yellow: p<.05. Genes that exhibited a p-value<.05 when compared with at least 50% of all other core genes are discussed in the text.
(0.56 MB TIF)

Table S1 Contents of the hotspots.
(0.14 MB DOC)
