# Supplementary figures and images for: Comparative ICE Genomics: Insights into the Evolution of the SXT/R391 Family of ICEs
Source: PLoS Genet. 2009 Dec 24;5(12):e1000786. doi: 10.1371/journal.pgen.1000786 (PMC2791158; doi:10.1371/journal.pgen.1000786)

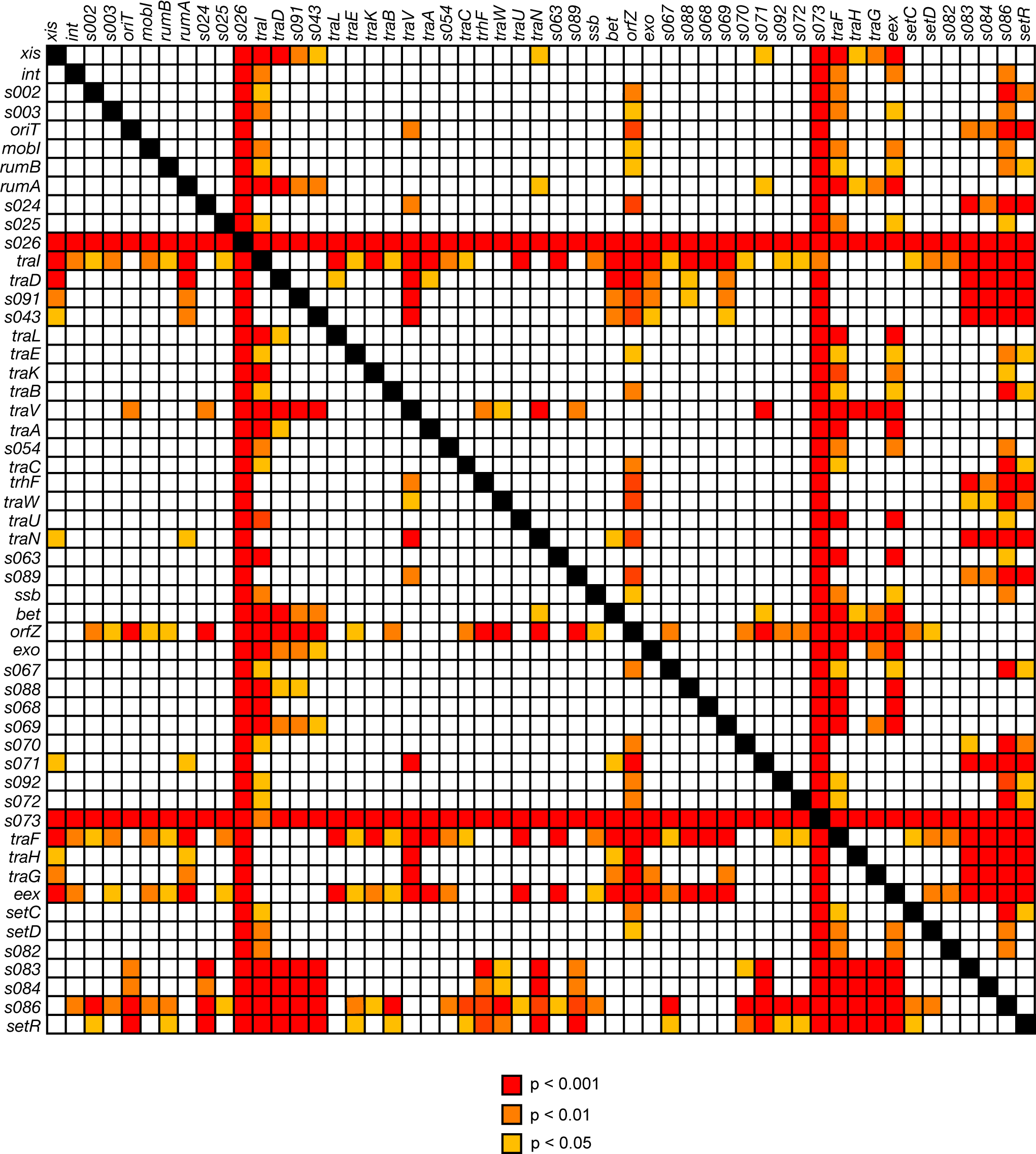

Supplement: Figure S1 — Variations in the conservation of individual core ICE genes. The percent identity of the nucleotide sequence of each core gene and oriT versus the corresponding sequence in SXT was calculated for all ICEs studied. The average values for each gene (as shown in the inset of Figure 5) were then used in one-way ANOVA comparisons to determine genes that exhibit significantly more or less conservation compared to other core genes. p-values of one-way ANOVA comparisons of each core ICE gene are shown. The grid represents all pair-wise comparisons, and the color indicates the level of significance as follows: red: p<.001, orange: p<.01, and yellow: p<.05. Genes that exhibited a p-value<.05 when compared with at least 50% of all other core genes are discussed in the text. (0.56 MB TIF) [file pgen.1000786.s001.tif]
