# Supplementary material for: Comparative ICE Genomics: Insights into the Evolution of the SXT/R391 Family of ICEs
Source: PLoS Genet. 2009 Dec 24;5(12):e1000786. doi: 10.1371/journal.pgen.1000786 (PMC2791158; doi:10.1371/journal.pgen.1000786)
Supplement: Table S1 — Contents of the hotspots. (0.14 MB DOC) [file pgen.1000786.s002.doc]

Table S1. Contents of the hotspots.

*Hotspot #1: traJ -* traL

| **ICE** | **Length (bp)** | **Number of ORFs** | **Hotspot Contents** |
| --- | --- | --- | --- |
| SXT | 676 | 2 | *s044, s045* (putative toxin-antitoxin) |
| ICE*Vch*Ind4 | 676 | 2 | *s044, s045* |
| ICE*Pal*Ban1 | 693 | 2 | *s044, s045* |
| R391 | 774 | 2 | *orf37*, *orf38* (conserved hypotheticals) |
| ICE*Pda*Spa1 | 744 | 2 | *orf37*, *orf38* |
| ICE*Vch*Ban5 | 774 | 2 | *orf37*, *orf38* |
| ICE*Vch*Ind5 | 774 | 2 | *orf37*, *orf38* |
| ICE*Pmi*Usa1 | 774 | 2 | *orf37*, *orf38* |
| ICE*Spu*PO1 | 774 | 2 | *orf37*, *orf38* |
| ICE*Vch*Moz10 | 774 | 2 | *orf37*, *orf38* |
| ICE*Vch*Ban9 | 774 | 2 | *orf37*, *orf38* |
| ICE*Vfl*Ind1 | 4039 | 6 | tnpC (integrase catalytic subunit), *vflind-1* (conserved hypothetical), *vflind-2* (Istb ATP-binding domain containing protein), *orf37*, *orf38* |
| ICE*Vch*Mex1 | 1333 | 2 | *mex01* (putative Fic family protein) |

*Hotspot #2: traA –* s054

| **ICE** | **Length (bp)** | **Number of ORFs** | **Hotspot Contents** |
| --- | --- | --- | --- |
| SXT | 1995 | 2 | *mosA*, *mosT* (toxin-antitoxin) |
| ICE*Vch*Ind4 | 1995 | 2 | *mosA*, *mosT* |
| ICE*Vch*Ind5 | 1995 | 2 | *mosA*, *mosT* |
| ICE*Pal*Ban1 | 1995 | 2 | *mosA*, *mosT* |
| ICE*Vch*Ban5 | 1995 | 2 | *mosA*, *mosT* |
| ICE*Pmi*Usa1 | 1994 | 2 | *mosA*, *mosT* |
| ICE*Vfl*Ind1 | 1992 | 2 | *mosA*, *mosT* |
| ICE*Pda*Spa1 | 9416 | 6 | *mosA, mosT, spa01* (putative helicase), *spa02*, spa03 (conserved hypotheticals), *spa04* (Fis family transcriptional regulator) |
| ICE*Vch*Moz10 | 9400 | 6 | *mosA*, *mosT*, *spa01*, *spa02*, *spa03*, *spa04* |
| ICE*Vch*Ban9 | 9400 | 6 | *mosA*, *mosT*, *spa01*, *spa02*, *spa03*, *spa04* |
| R391 | 6944 | 9 | *orf45* (putative N-acetyltransferase), *orf46*, *orf47*, *orf55* (conserved hypotheticals), *orf48* (IS4 family transposase), *orf50* (sulfate transporter), *orf52* (universal stress protein in family UspA), *orf53* (putative diguanylate cylcase), *orf54* (putative phosphodiesterase with PAS/PAC sensor) |
| ICE*Vch*Mex1 | 3120 | 4 | *orf45*, *orf46*, *orf47*, *mex02* (conserved hypothetical) |
| ICE*Spu*PO1 | 29210 | 25 | *sputw3181*, *sputw1110*, *sputw1115*, *sputw1126* (putative heavy metal translocating P-type ATPases), sputw*1111*, *sputw1112*, *sputw11120* (putative transcriptional regulators in MerR family), *sputw1113*, *sputw1117* (putative IstB ATP-binding domain containing proteins), sputw*1122*, *sputw1125* (putative transcriptional regulators in ArsR family) *sputw1123* (putative dihydrolipoamide dehydrogenase),  *sputw1124* (cation efflux family protein), sputw*1129*, *sputw1130*, (putative RND family efflux transporters), *sputw1131* (putative outer membrane efflux protein), *sputw1135a*, *sputw1135b* (putative efflux transporters HAE1), sputw*1136* (putative heavy metal transporter), sputw*1138* (conserved hypothetical), *tnp* (x5) |

*Hotspot #3: s073 -* traF

| **ICE** | **Length (bp)** | **Number of ORFs** | **Hotspot Contents** |
| --- | --- | --- | --- |
| SXT | 5526 | 2 | *s074* (putative response regulator), *s075* (putative two-component sensor histidine kinase) |
| ICE*Vch*Ind4 | 5527 | 2 | *s074*, *s075* |
| ICE*Vch*Mex1 | 9115 | 6 | *mex03*, *mex04* (conserved hypothetical), *mex05* (diguanylate cyclase with PAS/PAC domain), *cds1* (putative transposase), *s074*, *s075* |
| ICE*Vfl*Ind1 | 9115 | 6 | *mex03, mex04, mex05, cds1, s074, s075* |
| ICE*Vch*Moz10 | 4228 | 6 | *orfc5b* (putative antibiotic resistance/glyoxalase), *orfc5a* (AraC family transcriptional regulator), *orfc4*, *orfc2* (conserved hypotheticals), *orfc3* (membrane associated protein containing XRE-family DNA binding HTH domain), *intl9* (tyrosine recombinase) |
| ICE*Vch*Ban9 | 4810 | 7 | *orfc5b*, *orfc5a,* *orfc4*, *orfc3*, *orfc2*, *dfrA1* (trimethoprim resistance), *intl9* |
| ICE*Vch*Ind5 | 4810 | 7 | *orfc5b*, *orfc5a,* *orfc4*, *orfc3*, *orfc2*, *dfrA1*, *intl9* |
| ICE*Pal*Ban1 | 4810 | 7 | *orfc5b*, *orfc5a,* *orfc4*, *orfc3*, *orfc2*, *dfrA1*, *intl9* |
| ICE*Vch*Ban5 | 4810 | 7 | *orfc5b*, *orfc5a,* *orfc4*, *orfc3*, *orfc2*, *dfrA1*, *intl9* |
| R391 | 1419 | 1 | *orf80* (hypothetical protein) |
| ICE*Pmi*Usa1 | 4431 | 2 | *pmiHI4320-5* (putative exonuclease), *pmiHI4320-6* (putative UvrD/REP helicase) |
| ICE*Spu*PO1 | 9667 | 10 | *sputw3181*, *sputw1164*, *sputw1168*, *sputw1172* ( conserved hypotheticals), *sputw1165* (CzcA family heavy metal efflux protein), *sputw1166* (biotin/lipoyl attachment domain containing protein), *sputw1167* (outer membrane efflux protein), *sputw1169* (cation efflux system permease), *sputw1170* (MerR family transcriptional regulator), *sputw1171* (cation efflux family protein), |
| ICE*Pda*Spa1 | 10516 | 7 | *IS10*, *spa7* (heat shock protein Hsp70), *spa8* (AAA ATPase central-domain containing protein), *spa9*, *spa10*, *spa11*, *spa12* (conserved hypotheticals) |

*Hotspot #4: traN –* s063

| **ICE** | **Length (bp)** | **Number of ORFs** | **Hotspot Contents** |
| --- | --- | --- | --- |
| SXT | 3794 | 4 | *s060*, *s061* (conserved hypotheticals), *s062* (endonuclease I precursor), *s090* (conserved hypothetical) |
| ICE*Vch*Ind4 | 3787 | 4 | *s060*, *s061*, *s062*, *s090* |
| ICE*Vch*Ind5 | 6138 | 5 | *vchind5-9*, *vchind5-11* (SMC-like proteins), *vchind5-10* (IstA transposase), *tnp*, *s062* |
| ICE*Vch*Ban5 | 6138 | 5 | *vchind5-9*, *vchind5-11*, *vchind5-10*, *tnp*, *s062* |
| ICE*Spu*PO1 | 4113 | 4 | *sputw1146*, *sputw1147*, *sputw1148* (conserved hypotheticals), *s062* |
| ICE*Pmi*Usa1 | 4119 | 4 | *sputw1146*, *sputw1147*, *sputw1148*, *s062* |
| R391 | 1309 | 2 | *vchb33-4*, *orf64* (conserved hypotheticals) |
| ICE*Vch*Moz10 | 1310 | 2 | *vchb33-4*, *orf64* |
| ICE*Vch*Ban9 | 1310 | 2 | *vchb33-4*, *orf64* |
| ICE*Vfl*Ind1 | 1309 | 2 | *vchb33-4*, *orf64* |
| ICE*Pda*Spa1 | 1592 | 2 | *spa5*, *spa6* (conserved hypotheticals) |
| ICE*Pal*Ban1 | 7519 | 9 | *‘gntR* (putative transcriptional regulator in GntR family), *phzF* (phenazine biosynthesis protein PhzF family), *palban1-1* (conserved hypothetical), *palban1-2* (endoribonuclease L-PSP), *palban1-3* (putative YbaK/prolyl-tRNA synthetase family), *palban1-4* (maltose O-acetyltransferase, *palban1-5* (3-methyl-2-oxobutanoate hydroxymethyl transferase), *palban1-6* (lysine exporter protein), *palban1-7* (AsnC family transcriptional regulator) |
| ICE*Vch*Mex1 | 7575 | 4 | *uvrD* (UvrD helicase family), *top1* (putative endonuclease), *pept* (putative peptidase), *atpA* (AAA ATPase) |

*Hotspot #5: s026 -* traI

| **ICE** | **Length (bp)** | **Number of ORFs** | **Hotspot Contents** |
| --- | --- | --- | --- |
| SXT | 17966 | 9 | *s027*, *s028*, *s029*, *s031*, *s033*, *s040* (conserved hypotheticals), *s035* (restriction enzyme), *s038* (hypothetical protein with PglZ domain), *s039* (ATP-dependent Lon protease) |
| ICE*Vch*Ind4 | 17960 | 9 | *s027*, *s028*, *s029*, *s031*, *s033*, *s035*, *s038*, *s039*, *s040* |
| ICE*Vch*Ind5 | 14937 | 8 | *vchind5-1*, *vchind5-2*, *vchind5-3*, *vchind5-4*, *vchind5-7*, *vchind5-8* (conserved hypotheticals), *vchind5-5* (hypothetical protein with PglZ domain), *vchind5-6* (ATP-dependent Lon protease) |
| ICE*Vch*Ban5 | 14937 | 8 | *vchind5-1*, *vchind5-2*, *vchind5-3*, *vchind5-4*, *vchind5-7*, *vchind5-8*, *vchind5-5*, *vchind5-6* |
| ICE*Spu*PO1 | 13536 | 11 | *hsdM* (type I restriction modification system M subunit), *hsdS* (type I restriction modification system S subunit), *hsdR* (type III restriction enzyme res subunit), *sputw1089*, *sputw1091*, *sputw1092*, *sputw1093*, *sputw1094*, *sputw1095*, *sputw1096*, *sputw1097* (conserved hypotheticals) |
| ICE*Vfl*Ind1 | 13536 | 11 | *hsdM*, *hsdS* *, hsdR*, *sputw1089*, *sputw1091*, *sputw1092*, *sputw1093*, *sputw1094*, *sputw1095*, *sputw1096*, *sputw1097* |
| ICE*Pal*Ban1 | 13556 | 11 | *hsdM*, *hsdS*, *hsdR*, *sputw1089*, *sputw1091*, *sputw1092*, *sputw1093*, *sputw1094*, *sputw1095*, *sputw1096*, *sputw1097* |
| ICE*Pmi*Usa1 | 16933 | 4 | *pmiHI4320-1* (ATP-dependent helicase), *pmiHI4320-2* (conserved hypothetical), *pmiHI4320-3* (DEAD/DHAH helicase domain containing protein), *pmiHI4320-4* (UvrD/REP helicase) |
| R391 | 21496 | 15 | *tnpIS15* (x2), *orf17, orf18, orf22*, *orf27* (conserved hypotheticals), *kanR*, *tnp391A, s027*, *s028*, *s029*, *s035*, *s038, s039*, *s040* |
| ICE*Vch*Moz10 | 17872 | 8 | *s027*, *s028*, *s029*, *vchb33-3* (conserved hypothetical), *s035*, *s038*, *s039*, *s040* |
| ICE*Vch*Ban9 | 17872 | 8 | *s027*, *s028*, *s029*, *vchb33-3*, *s035*, *s038*, *s039*, *s040* |
| ICE*Pda*Spa1 | 19309 | 11 | *s027*, *s028*, *s029*, *s031*, *s033*, *s035* (x2), *IS10*, *s038*, *s039*, *s040* |
| ICE*Vch*Mex1 | 8205 | 6 | *hsdM*, *hsdS*, *fdp1* (Fic family protein), *hsdR*, ‘*mrr*, *mrr’* (restriction endonuclease proteins) |
